# Malpractice claims after antireflux surgery and paraesophageal hernia repair: a population-based analysis

**DOI:** 10.1007/s00464-023-10572-2

**Published:** 2023-11-27

**Authors:** Nelli M. J. Nurminen, Tommi K. M. Järvinen, Ville J. Kytö, Silja A. S. Salo, Caitlin E. Egan, Saana E. Andersson, Jari V. Räsänen, Ilkka K. P. Ilonen

**Affiliations:** 1 Department of General Thoracic and Esophageal Surgery, Heart and Lung Center, Helsinki University Hospital and University of Helsinki, Haartmaninkatu 4, 00290 Helsinki, Finland; 2 Turku Clinical Research Centre, Turku University Hospital, Turku, Finland; 3 Heart Center, Turku University Hospital and University of Turku, Turku, Finland; 4 Gastrointestinal Surgery, Abdominal Center, Helsinki University Hospital and University of Helsinki, Helsinki, Finland; 5 Weill Cornell Medicine, 1300 York Avenue, New York, NY 10065 USA; 6 Department of Surgery, Päijät-Häme Central Hospital, Lahti, Finland

**Keywords:** Paraesophageal hernia, Antireflux surgery, Malpractice claims, Complications

## Abstract

**Background:**

The complication rate of modern antireflux surgery or paraesophageal hernia repair is unknown, and previous estimates have been extrapolated from institutional cohorts.

**Methods:**

A population-based retrospective cohort study of patient injury cases involving antireflux surgery and paraesophageal hernia repair from the Finnish National Patient Injury Centre (PIC) register between Jan 2010 and Dec 2020. Additionally, the baseline data of all the patients who underwent antireflux and paraesophageal hernia operations between Jan 2010 and Dec 2018 were collected from the Finnish national care register.

**Results:**

During the study period, 5734 operations were performed, and the mean age of the patients was 54.9 ± 14.7 years, with 59.3% (n = 3402) being women. Out of all operations, 341 (5.9%) were revision antireflux or paraesophageal hernia repair procedures. Antireflux surgery was the primary operation for 79.9% (n = 4384) of patients, and paraesophageal hernia repair was the primary operation for 20.1% (n = 1101) of patients. A total of 92.5% (5302) of all the operations were laparoscopic. From 2010 to 2020, 60 patient injury claims were identified, with half (50.0%) of the claims being related to paraesophageal hernia repair. One of the claims was made due to an injury that resulted in a patient's death (1.7%). The mean Comprehensive Complication Index scores were 35.9 (± 20.7) and 47.6 (± 20.8) (p = 0.033) for antireflux surgery and paraesophageal hernia repair, respectively. Eleven (18.3%) of the claims pertained to redo surgery.

**Conclusions:**

The rate of antireflux surgery has diminished and the rate of paraesophageal hernia repair has risen in Finland during the era of minimally invasive surgery. Claims to the PIC remain rare, but claims regarding paraesophageal hernia repairs and redo surgery are overrepresented. Additionally, paraesophageal hernia repair is associated with more serious complications.

Laparoscopic fundoplication is considered the standard surgical treatment for patients with gastroesophageal reflux disease (GERD). Antireflux surgery (AS) has been shown to improve symptoms of GERD and is indicated for a very selected patients who continue to have objectively demonstrated reflux-related symptoms despite receiving optimal pharmacological therapy or as an alternative to it [1–4]. Studies have questioned the long-term effectiveness of AS compared to medical therapy with proton pump inhibitors (PPIs), and the rate of AS has declined substantially, but it is still commonly performed [5–9]. Fundoplication is also an integral part of paraesophageal hernia repair (PEHR), the incidence of which has reportedly increased [10]. Operative indications of paraesophageal hernias are not as clear as for AS, but incarceration or symptomatic type two, three, or four hiatal hernias are the most well-established reasons to perform PEHR [11]. The objective of fundoplication in both treatment indications is to prevent reflux of gastric contents to the esophagus by reconstructing the gastroesophageal junction and to anchor the stomach below the hiatus.

Both AS and PEHR pose risks for perioperative and postoperative complications that may cause morbidity in both the short and long term. Specific complications of AS and PEHR include iatrogenic esophageal or gastric perforation, dysphagia, gas-bloat syndrome, and vagal nerve injury [8, 12–15]. Both operations are associated with a significant treatment failure rate, as the risks of recurrent GERD symptoms and hernia are 17.7% and 5–25.5% respectively [8, 16, 17]. The reasons and risk factors for complications or treatment failure are not completely understood. A recent population-based study from the Nordic Antireflux Surgery Cohort found that high hospital volume was not associated with a decreased risk of reintervention after antireflux surgery [18].

Reviewing malpractice claims can provide real-life patient-centric data on adverse events and potentially improve patient safety [19]. Although not synonymous, complications can also be investigated through malpractice claims. Additionally, malpractice claims in Finland are patient-initiated and thus reflect complications that matter to the patients. They can also offer an important perspective on the possible legal repercussions of complications for surgeons.

In a previous historical national report of AS and PEHR in Finland from 1992 to 2001, 37% of operations were open and 63% laparoscopic, the 30-day mortality rate was 1.0 per 1000 operations, and the annual number of operations increased from 600 to 1400 during the study period [20]. Following this study, patient selection changed due to the increased use of PPIs, and more comprehensive preoperative diagnostics, such as high-resolution manometry, have become commonplace [21]. In a more recent study from the Nordic Antireflux Surgery Cohort, published in 2021, the 90-day mortality rate was 0.13% [22].

The primary aim of this national registry study was to gain insight about the rates of mortality and malpractice claims of both AS and PEHR in a population-based setting, with respect to the advent of minimally invasive surgery, using data from Finnish national databases. Second, with available data regarding malpractice claims, a more comprehensive analysis of major complications and long-term morbidity can be conducted. Finally, we aimed to compare these results to previously reported patient outcomes.

## Materials and methods

National AS and PEHR patient cohort data were obtained from the care register of the Finnish Institute for Health and Welfare between Jan 1st, 2010 and Dec 31st, 2018 [23]. All hospitals in Finland are mandated to annually report the details of every patient and the care given to the care register, with a reporting accuracy of 75–99% for common diagnoses [24]. Operations and procedures were reported using the Nordic Medico-Statistical Committee’s (NOMESCO) Classification of Surgical Procedures. For this cohort, cases involving AS and PEHR were identified using NOMESCO codes for AS and PEHR procedures (JBC00, JBC01, JBB00, JBB01, JBB90, JBB92, JBB91, and JBB93). From the Finnish Care Register data, we were also able to formulate a Charlson Comorbidity Index (CCI) for each patient [25]. For these patients, mortality up to 90 days was calculated from Finland’s national statistics on causes of death.

Additionally, data from all adult malpractice claims for AS and PEHR operations between Jan 1st, 2010 and Dec 31st, 2020 were collected from the national Patient Injury Centre (PIC) registry using the previously mentioned NOMESCO codes. Parallel to the judicial system, Finland uses a no-fault patient insurance system, which results in low-threshold reporting and the claiming of compensation without any legal advice. The PIC handles all the claims filed from both public and private health care systems, decides if an injury is compensable, and pays compensation accordingly. Patient insurance in Finland and the duties of the PIC are laid down in the Patient Insurance Act (948/2019), which also mandates that all care providers must have patient insurance. The PIC holds a national database of all filed patient injuries, including applicable patient records and the outcome of the claim. The most common criterion for a claim to be deemed compensable is its preventability: The PIC experts evaluate whether an experienced medical professional could have avoided the injury by choosing a different course of action [26, 27].

When analyzing PIC data, cases were separated into two categories. Those without a hiatal hernia and those with a type I hiatal hernia, which were classified as AS. Cases with type II, III, or IV hiatal hernias were classified as PEHR. Both AS and PEHR were included in the analysis due to similarities in their operations. However, the distinction was made because the decision-making process for whether to operate is very different for AS and PEHR.

A CCI was calculated for each patient. Records of all postoperative complications involved were collected, the complications were classified by the Clavien–Dindo classification for complications, and a Comprehensive Complication Index was calculated for each patient [28, 29].

Statistical analyses were done using SPSS Statistics version 27.0.1.0 (IBM Corp, Armonk, NY, USA) and SAS version 9.2 (SAS Institute, Cary, NJ, USA). A chi-square test was used to compare results with categorial variables and a Student’s *t*-test was used to compare continuous variables. Two-tailed p values under 0.05 were considered statistically significant. Logistic regression was used to study the associations of age, sex, year of operation, CCI, center volume, and surgery type (open or laparoscopic) with 30-day mortality.

This study is reported in line with the Strengthening the Reporting of Cohort Studies in Surgery (STROCCS) statement criteria [30]. As a retrospective registry study, no approval from an ethics committee was needed.

## Results

A total of 5734 AS and PEHR operations were performed during the study period. The mean age of the patient population was 54.9 ± 14.7 years and 59.3% (n = 3402) of the patients were women. The CCI was 0 for 58.6% (n = 3160), 1–2 for 34.6% (n = 1866) and ≥ 3 for 6.8% (n = 367) of patients. Overall, 432 (7.5%) operations were open and 5302 (92.5%) were laparoscopic. The intraoperative conversion rate was 0.56% (n = 30).

**Fig. 1:**
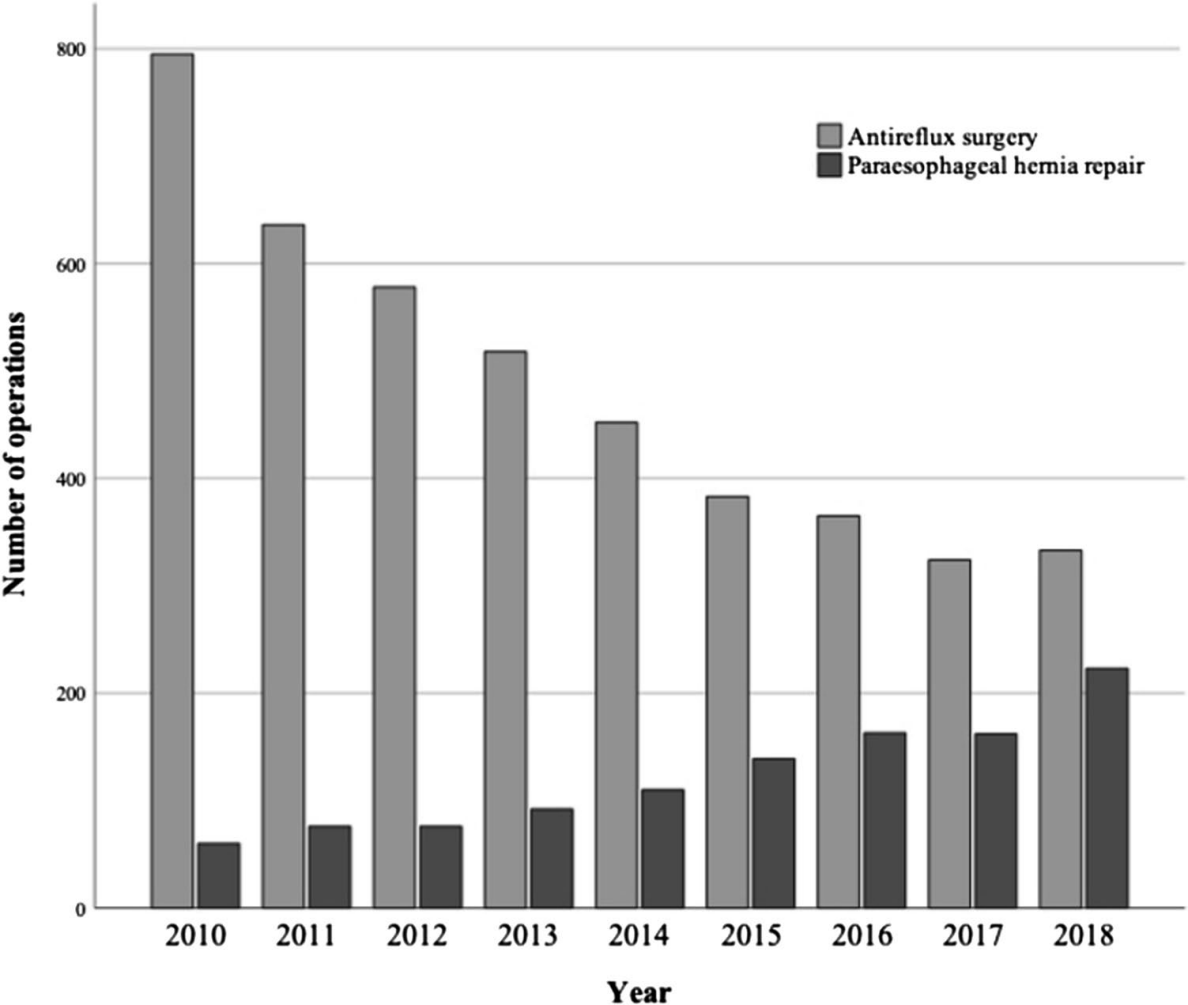
Trends in antireflux surgery and paraesophageal hernia repair in Finland during the period 2010–2018

To estimate the number of reoperations, we identified 341 (5.9%) patients who underwent multiple AS or PEHR operations within the study period. Twenty-five (0.4%) patients had three or more operations. The index operation was used to evaluate patient comorbidities and was used in the comparative studies for statistical accuracy. Of these, 79.9% (n = 4384) were coded as AS, 20.1% (n = 1101) as PEHR, and 1.7% (n = 92) were coded as both. Annual rates of AS and PEHR are shown in Fig. 1.

The 30-day and 90-day all-cause mortality rates were 0.45% (n = 24) and 0.61% (n = 33), respectively. The 30-day mortality was 0.23% (n = 19) for AS and 1.36% (n = 15) for PEHR. The 90-day mortality was 0.27% (n = 12) for AS and 2.1% (n = 23) for PEHR. Of the patients who underwent PEHR operations, 12.8% (n = 200) had an ICD-10 code related to incarceration (K44.0) or necrosis (K44.1) of a hiatal hernia.

A total of 36 centers were identified, 11 (30.6%) of which had an annual volume of over 20 AS and PEHR operations, 11 (30.6%) performed 8 to 20 AS and PEHR operations annually, and 14 (38.9%) performed fewer than eight AS and PEHR operations annually. Of the operations, 68.8% (n = 3708) were performed in centers with a volume of over 20 AS and PEHR operations, 24.5% (n = 1320) in centers that had a volume of 8 to 20 AS and PEHR operations, and 6.8% (n = 365) in centers with fewer than eight AS and PEHR operations annually. Regional central hospitals performed 2656 (46.3%) of the operations, whereas university hospitals performed 2394 (41.8%) and other hospitals performed 684 (11.9%).

Logistic regression analyses for preoperative factors are presented in Table 1. Older age, CCI over 3, and open surgery are associated with higher mortality.

**Table 1:** Association of age, Charlson Comorbidity Index, and the approach of surgery with 30-day mortality

	30-day mortality (%)	Univariable			Multivariable		
		OR	95% CI	*p*	OR	95% CI	*p*
Age (per 1 year increment)		1.11	1.07, 1.15	0.001	1.08	1.04, 1.13	0.001
Sex							
Female	0.50	Ref.			Ref.		
Male	0.36	0.72	0.31, 1.68	0.446	0.81	0.33, 1.98	0.647
Charlson Comorbidity index				0.001			0.001
0	0.16	Ref.			Ref.		
1	0.30	1.91	0.51, 7.13	0.162	1.06	0.27, 4.12	0.205
2	0.55	3.51	0.84, 14.74	0.975	1.32	0.30, 5.92	0.501
≥ 3	3.27	21.33	7.47, 60.89	0.001	8.08	2.62, 24.93	0.001
Year of surgery							
2010–2013	0.39	Ref.			Ref.		
2014–2018	0.50	1.28	0.57, 2.85	0.553	1.00	0.42, 2.35	0.991
Center volume per year				0.164			0.025
< 8	0.82	Ref.			Ref.		
8–20	0.15	0.18	0.03, 1.10	0.059	0.07	0.01, 0.48	0.011
> 20	0.51	0.62	0.18, 2.11	0.466	0.31	0.08, 1.17	0.774
Surgical approach							
Laparoscopic	0.28	Ref.			Ref.		
Open	2.79	10.31	4.55, 23.37	0.001	6.73	2.75, 16.45	0.001

### Patient injury claims

From the PIC database, 61 patient injury claims related to AS and PEHR were identified. One claim included an operation in which a Heller myotomy was also performed, and this case was therefore excluded from the analysis. The mean age at the time of index surgery for patients who had filed claims was 57.2 years (SD ± 12.4, min–max 32–82 years) and 39 (65.0%) of the patients were women. In 50.0% (n = 30) of claims the patient did not have a hiatal hernia or had a type one hiatal hernia and this was therefore considered AS in the analysis. In the other 50.0% (n = 30) of claims, the patient had type three (n = 22, 36.7%) or type four (n = 8, 13.3%) hiatal hernia, and this was considered PEHR. None of the patients had type two hiatal hernias. The characteristics of the patients are described in more detail in Table 2.

**Table 2:** Preoperative characteristics of patients concerning patient injury claims resolved in the period 2010–2020 in the Finnish Patient Injury Centre database

	AS (no hiatal hernia or type 1) n = 30 Mean ± SD or % (n)	PEHR (hiatal hernia type 3 or 4) n = 30 Mean ± SD or % (n)	p
Age, years	52 ± 12	63 ± 10	0.001
Sex			0.589
Female	60 (18)	70 (21)	
Male	40 (12)	30 (9)	
Charlson Comorbidity Index			
0	70 (21)	47 (14)	0.115
≥ 1	30 (9)	53 (16)	
Prior abdominal surgery			0.606
No	43 (13)	53 (16)	
Yes	57 (17)	47 (14)	
Preoperative workup			
Upper endoscopy			0.103
Yes	97 (29)	80 (24)	
No	3 (1)	20 (6)	
pH testing			0.001
Yes	43 (13)	0 (0)	
No	57 (17)	100 (30)	
Manometry			0.001
Yes	15 (15)	7 (2)	
No	50 (15)	93 (28)	
Computer tomography			0.001
Yes	14 (4)	63 (19)	
No	26 (87)	37 (11)	

*AS* antireflux surgery, *PEHR* paraesophageal hernia repair

Of all claims, 60.0% (n = 36) concerned high-volume hospitals (annually over 20 operations per year), 18.3% (n = 11) intermediate volume hospitals (annually 8 to 20 operations), and 10.0% (n = 6) low-volume hospitals (annually fewer than eight operations). Furthermore, 11.7% (n = 7) of procedures were performed outside of the public healthcare system. None of the operations involved Collis gastroplasty, and none were robot-assisted. The characteristics of the operations are described in Table 3.

**Table 3:** Perioperative characteristics of patients concerning patient injury claims resolved in the period 2010–2020 in the Finnish Patient Insurance Centre database

	AS n = 30 Mean ± SD or % (n)	PEHR n = 30 Mean ± SD or % (n)	p
Hospital			0.110
Academic	27 (8)	50 (15)	
Other	73 (22)	50 (15)	
Hospital volume^a^			0.002
< 8	17 (5)	3 (1)	
8–20	10 (3)	27 (8)	
> 20	50 (15)	70 (21)	
Unknown	23 (7)	0 (0)	
Surgical approach			0.319
Laparoscopic	90 (27)	87 (26)	
Laparotomy	10 (3)	3 (1)	
Thoracotomy	0 (0)	10 (3)	
Intraoperative conversion	10 (3)	13 (4)	
Fundoplication type			0.409
Nissen	93 (28)	80 (24)	
Partial fundoplication	3 (1)	13 (4)	
None or gastropexy	3 (1)	7 (2)	
Hiatal mesh repair	0 (0)	20 (6)	0.024
Reoperation	23 (7)	13 (4)	0.506
Urgent surgery	0 (0)	7 (2)	0.492

*AS* antireflux surgery, *PEHR* paraesophageal hernia repair

^a^Annual volume of AS and PEHR operations in a hospital

Of the claims, 93.3% (n = 56) involved at least one identifiable complication and 61.7% (n = 37) involved several complications. One claim in the PEHR group involved an injury that led to the patient’s death (1.7%); the patient had acute paraesophageal hernia and perforation. Four claims (6.6%) did not involve any complications, but the patients were unsatisfied with how they were treated or still had residual symptoms. The characteristics of the complications are described in Table 4.

**Table 4:** Summary of adverse events of patients concerning patient injury claims resolved in the period 2010–2020 in the Finnish Patient Insurance Centre database

	AS n = 30 Mean ± SD or % (n)	PEHR n = 30 Mean ± SD or % (n)	p
Perforation	23 (7)	33 (10)	0.567
Esophageal	3 (1)	23 (7)	0.052
Gastric	13 (4)	7 (2)	0.067
Intestinal	10 (3)	3 (1)	0.061
Re-herniation	17 (5)	27 (8)	0.532
Early^a^	13 (4)	13 (4)	
Late	3 (1)	13 (4)	
Dysphagia	23 (7)	10 (3)	0.299
Bleeding	10 (3)	3 (1)	0.612
Splenectomy	7 (2)	0 (0)	0.492
Vagal nerve injury	7 (2)	3 (1)	1.000
Loss of esophageal continuity	0 (0)	7 (2)	0.492
Long-term disability^b^	17 (5)	17 (5)	1.000
No identified complications	10 (3)	3 (1)	0.612

*AS* antireflux surgery, *PEHR* paraesophageal hernia repair

^a^Re-herniation that occurred in the hospital or within 30 days of the index operation

^b^Severe symptoms lasting over 6 months

We classified all the identified complications using the Clavien–Dindo classification system and the Comprehensive Complication Index was calculated for each patient. The highest Clavien–Dindo grade was I for 3.3% (n = 2), II for 13.3% (n = 8), IIIa for 3.3% (n = 2), IIIb for 46.7% (n = 28), IVa for 20.0% (n = 12), IVb for 4.9% (n = 3), and V for 1.7% (n = 1) of the claims. Forty-four (73.3%) patients had a complication that led to reoperation under general anesthesia or needed intensive care. Of the patients who needed intensive care (n = 16, 26.7%) and of those who did not, the mean ages were 64.19 ± 12.5 and 54.68 ± 11.6 years respectively (*p* = 0.008). Sixty-five percent (n = 39) of patients had to undergo reoperation, and 20% (n = 12) had several reoperations. Of the first reoperations, 17 were laparoscopies, 15 were laparotomies, and 7 were thoracotomies. The mean Comprehensive Complication Index was 41.8, SD ± 21.2. For patients who underwent AS the mean Comprehensive Complication Index was 35.9 (± 20.7), and for patients who underwent PEHR the mean Comprehensive Complication Index was 47.6 (± 20.8) (*p* = 0.033). Patients who underwent redo surgery (n = 11, 18.3%) had a higher mean Comprehensive Complication Index (47.7, SD ± 7.6) than the others (n = 49, 81.7%, mean Comprehensive Complication Index 40.4, SD ± 2.9), but the difference was not statistically significant (*p* = 0.390).

Ten (16.7%) patients had a complication that resulted in long-term (> 6 months) symptoms or disability. Of these, four patients had dysphagia that needed repeated endoscopic dilatations or tube feeding for more than 6 months. Two patients needed esophagectomy with a spit fistula due to an iatrogenic perforation. Two patients had vagal nerve injury, resulting in long-lasting symptoms. One patient had early recurrent herniation that led to cardiac arrest and resuscitation, resulting in severe brain damage. One patient had an empyema that needed operative intervention via thoracotomy, which then led to chronic pain and long-term disability.

Twenty-two (36.7%) of the claims received were compensated. Half of the compensated claims concerned AS (n = 11), and half concerned PEHR (n = 11).

## Discussion

Between 2010 and 2018, 5734 AS and PEHR procedures were performed in Finland, resulting in 53 PIC claims, with an incidence of 1/1000 operations/year. Additionally, 7 claims were filed from 2019 to 2020 and analyzed. PIC claims were mostly filed after severe complications and comprised 50% AS and PEHR. By comparing the baseline data, PEHR and redo surgeries were overrepresented, and PEHR and AS showed different complication profiles, with patients filing claims after PEHR having a higher Comprehensive Complication index.

Comparing previous national reports of AS and PEHR, the combined rate of AS and PEHR decreased from 1400 operations in 2001 to 611 operations in 2018 [20]. In contrast to these reported numbers, the rate of PEHR increased during our study period from 60 operations in 2010 to 223 operations in 2018. Additionally, compared to the earlier national report on PEHR from 2001, the mean rate of AS and PEHR operations was 37.8 operations annually, whereas in our study, the mean rate was 137.6 operations per year [31]. These trends are in line with other studies of different populations [5, 6, 32]. The increase in PEHR might be explained by the increased usage of laparoscopy in more complex cases, as laparoscopic PEHR has less associated morbidity than open surgery and has been shown to be safe for the elderly and patients with comorbidities [33–39]. The increase in popularity of PEHR may also be related to the rapidly increasing proportion of aging adults in the population population and increase in computed tomography imaging [40, 41].

In our cohort, the all-cause mortality rate after AS was low and comparable to that in similar studies [5]. The 30-day and 90-day mortality after PEHR were higher—1.36% and 2.1% respectively. This may be due to cases requiring urgent and emergency PEHR, which are known to bear a much higher mortality [42, 43]. Although other studies have stated that laparoscopic PEHR is safe in elderly patients, our multivariate regression model and univariate analysis of claim data showed that older age was associated with higher mortality and morbidity rates [44, 45].

Compared to the baseline cohort, the patients in the PIC group were slightly older and underwent more PEHRs. Of the operations described in the claims concerning PEHR, 70% had been performed in high-volume centers, which is thought to be associated with better outcomes [46]. Emergency PEHR operations were underrepresented in the PIC group, as only 6.7% were for emergency PEHR, whereas in the population data, emergency PEHR was involved in 12.8% of cases.

In the PIC patient group, reoperations were overrepresented, as 18.3% of the claims dealt with reoperations, whereas 5.9% of all patients had one or more reoperation after the index operation. This is in line with the notion that although considered to be safe and feasible, redo AS or PEHR has higher morbidity and mortality rates than primary operations [47, 48]. This highlights the importance of patient selection and informing patients of increased risks of adverse events, especially when considering reoperation.

Most PIC claims dealt with objective severe complications in both the AS and PEHR patient groups (Table 4). The patients who filed claims concerning PEHR had a significantly higher mean Comprehensive Complication Index than those who filed claims concerning AS. This can be attributed to increased frailty due to aging, as the PEHR group was older [49]. Additionally, the hiatus was reinforced with a mesh in 20% of patients in the PEHR group, which a recent meta-analysis by Angeramo et al. found to be associated with a higher overall morbidity rate than repair with only sutures [50]. Although the differences were not statistically significant, the profile of complications was different in the AS and PEHR groups. Perforations occurred more during PEHR than AS. This is in line with a previous institutional study by Zhang et al. consisting of 1223 foregut surgeries [15]. Dysphagia was more common in the AS group, although the difference from the PEHR group was not statistically significant. This might be explained by the higher rate of partial fundoplications in the PEHR group, as they are associated with less dysphagia (Table 3) [12, 51].

In the AS patients within the PIC group, preoperative diagnostic testing was not performed according to current guidelines, as only 43.3% of the claimants had pH surveillance done preoperatively (Table 2); although this has been recommended since the 1990s. [1, 4, 52] Also, preoperative manometry was performed only in 50.0% of AS patients in the PIC patient group (Table 2). These findings do not explain the observed adverse events, but they put the justification of the operation into doubt. Adherence to guidelines for the baseline cohort is unknown. In the randomized trial by Spechler et al., 79% of the enrolled patients did not meet the criteria for surgery, thus calling the extent of workup needed to find good candidates for antireflux surgery into question [2].

In previous literature examining the relationship between surgeon or hospital volume and patient outcomes generally favors higher volume [18, 53, 54]. In our data regarding all operations, 30-day mortality was lowest in medium-volume hospitals (8–20 per year), which can be attributed to selection bias, as more complex cases are referred to academic and other high-volume hospitals. Most of the PIC claims involved operations performed in high-volume hospitals, which is most likely due to the same bias.

Our study has several limitations. First, we were not able to obtain baseline data for all AS and PEHR operations performed from 2019 to 2020 due to changes in laws concerning the secondary use of health and social data. Second, as for all retrospective analyses, ours is prone to selection bias and, therefore, cannot conclude causality. Third, the Care Registry does not provide information on potentially significant confounding factors like obesity, smoking status, and prior abdominal surgery. Additionally, we were only able to assess the volume of the hospitals, not the volume of single surgeons. Additionally, in Care Registry data, AS and PEHR procedures are differentiated based on different NOMESCO codes instead of the actual sizes of the paraesophageal hernias so some cases may be miscoded. We were only able to report overall mortality, not disease-specific mortality. Although the threshold to file a claim is low within the national PIC system, it is patient-driven, and therefore not all complications are reported. PIC data may also be affected by sampling bias, which might reduce the generalizability of the results [55]. From the patient injury data, we could not assess the severity of symptoms, usage of PPIs, or quality of life. Because of the timeline of the claim process, some claims concerning operations performed during our study time may not have been filed before these data were collected.

This study highlights a significant change in the trends of foregut surgery, with the focus shifting from AS to PEHR at the national level. This transition is also evident in the number of malpractice claims, with relatively more claims associated with PEHR due to the complexity of the surgery, older patient age, and medical comorbidities. Nevertheless, both AS and PEHR are generally safe during the era of minimally invasive surgery, and patient injury claims are rare. As the aims of both procedures are to alleviate symptoms and improve quality of life, the occurrence of severe complications should be extremely low.
